# Mutagenicity of drinking water sampled from the Yangtze River and Hanshui River (Wuhan section) and correlations with water quality parameters

**DOI:** 10.1038/srep09572

**Published:** 2015-03-31

**Authors:** Xuemin Lv, Yi Lu, Xiaoming Yang, Xiaorong Dong, Kunpeng Ma, Sanhua Xiao, Yazhou Wang, Fei Tang

**Affiliations:** 1 Department of Environmental Microbiology, Institute of Environmental Medicine, MOE Key Lab of Environment and Health, School of Public Health, Tongji Medical College, Huazhong University of Science and Technology, Wuhan 430030, PR China

## Abstract

A total of 54 water samples were collected during three different hydrologic periods (level period, wet period, and dry period) from Plant A and Plant B (a source for Yangtze River and Hanshui River water, respectively), and several water parameters, such as chemical oxygen demand (COD), turbidity, and total organic carbon (TOC), were simultaneously analyzed. The mutagenicity of the water samples was evaluated using the Ames test with *Salmonella typhimurium* strains TA98 and TA100. According to the results, the organic compounds in the water were largely frame-shift mutagens, as positive results were found for most of the tests using TA98. All of the finished water samples exhibited stronger mutagenicity than the relative raw and distribution water samples, with water samples collected from Plant B presenting stronger mutagenic strength than those from Plant A. The finished water samples from Plant A displayed a seasonal-dependent variation. Water parameters including COD (*r* = 0.599, *P* = 0.009), TOC (*r* = 0.681, *P* = 0.02), UV_254_ (*r* = 0.711, *P* = 0.001), and total nitrogen (*r* = 0.570, *P* = 0.014) exhibited good correlations with mutagenicity (TA98), at 2.0 L/plate, which bolsters the argument of the importance of using mutagenicity as a new parameter to assess the quality of drinking water.

Surface waters, such as rivers and lakes, receive large amounts of waste water from industrial, agricultural, and domestic sources as well as from municipal sewage treatment plants. These waters, which contain unidentified substances, are typically used as the source of drinking water. Chlorination has been widely applied in the disinfection process of drinking water for decades and can effectively reduce waterborne infections caused by pathogenic microorganisms^1^; and the DBPs were concerned and detected since the 1970s^2^,^3^,^4^. Many researchers have reported that these types of DBPs present mutagenic, genotoxic, and carcinogenic effects in *in vitro* and *in* *vivo* experiments^5^,^6^. In addition, many epidemiological studies have indicated that exposure to these compounds is highly related to the occurrence of bladder and rectal cancer, growth retardation, spontaneous abortion, and congenital cardiac defects^7^,^8^,^9^.

The water environment has deteriorated over the past 30 years. Indeed, many researchers have reported that substances with genotoxic, and mutagenic activities are detectable in most source waters in China^5^,^10^,^11^,^12^. The Yangtze River, the largest river in Asia, with a length of 6,397 km, flows through Wuhan City, the capital of Hubei Province, with a population of 10,000,000, and is the main water supply for the city. The Hanshui River, the largest tributary of the Yangtze River, with a length of 1,577 km and 75% catchment areas in Hubei Province, rises from Hanzhong City and flows through the Jianghan Plain and empties at Wuhan City into the Yangtze River. In Wuhan City, the water quality of the Yangtze River is mainly affected by the Hanshui River, and both rivers are used as the main water source for the city. Ten years ago, Yuan et al.^13^ investigated the genotoxic potential of water from the Yangtze River and Hanshui River in Wuhan and found that all water extracts caused dose-dependent DNA mutation at certain concentrations. Additional studies have noted that in the water system of the Yangtze River basin, such as Taihu Lake and Yangtze Delta, mutagenicity varies during different seasons and that different treatment processes result in various mutagenic strengths^14^,^15^. Because of the many sewage outfalls along their banks, the water quality of the Yangtze River and Hanshui River is threatening the health of more than a hundred million people. However, few reports have been published on the genotoxic potential and the seasonal-dependent variations in the genotoxicity of the water in the two rivers.

Currently, qualitative and quantitative analyses of drinking water generally involve chromatography and other methods; however, the equipment required is expensive, and measuring the trace level of organic compounds in water requires complex pretreatment, which hampers routine monitoring. Furthermore, chemical analysis cannot completely evaluate exposure to various organic toxins and therefore cannot reflect joint toxic effects^16^,^17^. Toxicity assays of an entire sample are important for improving the assessment of hazardous chemicals in drinking water^18^. In the USA, some bioassays are applied for acute or chronic toxicity detection and can be used as a criterion for water quality^19^. Similarly, in China, the application of genetic toxicology assays in aquatic system monitoring is necessary and feasible^15^. Evaluating organic compounds in drinking water usually involves the chemical oxygen demand (COD), biochemical oxygen demand (BOD), total organic carbon (TOC), ammonia nitrogen, nitrate nitrogen, total nitrogen, and other water quality parameters, but these parameters fail to evaluate in detail the contents of toxic pollutants and the strength of their biological toxicity. Therefore, correlations between water quality parameters and biological effects need to be demonstrated. Indeed, the application of water quality parameters to quickly and economically predict the biological toxicity strength of organic pollutants in water has become an issue of concern for both domestic and foreign research organizations^20^,^21^.

The objectives of this study were to (a) further investigate the mutagenic activity of organic contamination in the Yangtze River and Hanshui River, (b) explore the correlations between the water quality parameters and mutagenicity strength, and (c) recommend mutagenicity data as a new parameter to assess the quality of drinking water.

## Methods

### Chemicals

Chemicals were obtained as follows: glucose-6-phosphate, nicotinamide adenine dinucleotide phosphate (NADP), 2-aminofluorene (2-AF), dimethyl sulfoxide (DMSO), L-histidine, and sodium p-(dimethylamino)benzenediazo sulfonate (Dexon) from the Sigma Chemical Co. (USA); Amberlite XAD-2 resin from Serva Chemical Co. (Germany); and acetone, dichloromethane, and other chemicals of analytical grade from Wuhan Chemical Co. (China).

### Water sample collection and SPE extraction

**Figure 1:**
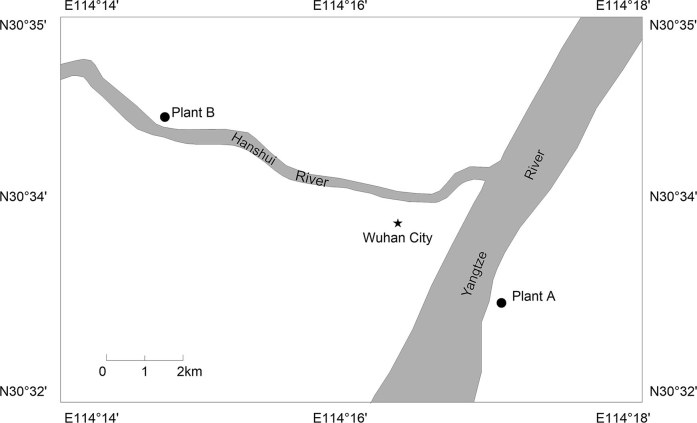
The Location of Sampling Points along the Yangtze River and Hanshui River. Plant A: source of Yangtze River water. Plant B: source of Hanshui River water.

From 2007 to 2009, a total of 54 water samples, including raw water, finished water, and distribution water, from three different hydrologic periods (level period (March), wet period (July), and dry period (December)) were collected from the two water plants using stainless steel drums. As illustrated in Figure 1, Plant A, located at the center of the city, receives water from the Yangtze River and supplies the most populated and concentrated areas. Plant B is supplied by water from the Hanshui River and is located at an estuary; thus, the water samples collected there should represent the water quality of the Yangtze River. Both plants apply a conventional water treatment process that includes prechlorination, coagulation, sedimentation, filtration, and chlorination, similar to the description by Wang^22^.

Each sample was approximately 140 L. The organic compounds in the water samples were extracted using XAD-2 resin, and the columns (40 mL) were cleaned and conditioned following the standard methods^23^. In brief, the columns were conditioned with methanol, ether, and acetonitrile in a Soxhlet extractor for more than 8 hours. As the finished water and distribution water were relatively clean, they were directly percolated through the columns, whereas the raw water samples were filtered through a 1-μm glass fiber filter to separate the dissolved and particulate phases prior to extraction^24^. Then, all water samples were percolated through the columns at a flow rate of 40 mL/min within 12 h of collection. The columns were then washed with 100 mL tri-distilled water and then dried under vacuum for 1 h. Subsequently, the retained analytes were eluted at a flow rate of 10 mL/min with acetone (40 mL) and an acetone:n-hexane solution (120 mL, 25:75, v/v), followed by 80 mL dichloromethane. Blanks were included to confirm the absence of carryover. The eluents were then reduced to 10 mL using a vacuum rotary evaporator in a water bath at 40°C. Finally, the samples were dried under a gentle nitrogen stream. For the Ames test, these extracts were dissolved in DMSO^25^,^26^ and were matched into a specific concentration series to make 0.1 mL solutions containing organic compounds from 0.5 L, 1 L, and 2 L water to produce 0.5, 1.0, and 2.0 L equivalent/plate of water samples, respectively. The concentrates were stored at −20°C for further analysis.

It should be pointed out that during the water sample treatment described above, most of the volatile organic compounds will not be absorbed. Our aim is focusing on the nonvolatile groups for in China and most of the countries around the world, people boil water before drinking, and many studies pointed out that volatile DBPs like chloroform and other THMs are not mutagenic or genotoxic in a wide array of system and end point *in vivo* and *in vitro*^27^,^28^,^29^. Thus in this study we mainly focus on the nonvolatile groups.

### Chemical analysis

Water parameters, including chemical oxygen demand (COD), residual chlorine, ammonia nitrogen, total nitrogen, nitrate, total organic carbon (TOC) and UV_254_, were detected following the national standard methods for drinking water quality of China^30^, turbidity, pH, and water temperature were obtained from the water quality monitoring department at each water plant (Supplementary Tables S1, S2, and S3).

### Ames test

The *Salmonella*/microsome mutagenicity assay is a short-term bacterial assay specifically designed to detect substances that can cause genetic damage that leads to mutations and to explore the mutagenic potential of mixtures^31^. As the Ames test is widely used as an initial screen to determine the mutagenic potential of water samples, the mutagenicity of the extracts of water samples taken from the Yangtze River and Hanshui River in this study was assessed using the *Salmonella* bioassay.

*Salmonella typhimurium* strains TA98 and TA100 were kindly provided by Prof. B.N. Ames (University of California, Berkley, USA) and stored at −80°C. The procedures for bacterial culture, identification of genetic characters, and plate incorporation assay were essentially the same as described by Maron and Ames^31^,^32^. 2-AF (20 μg/plate) and Dexon (50 μg/plate) were used as positive controls with and without S9 (a microsomal enzyme metabolic activation mixture); DMSO served as the negative solvent control. All determinations were performed in triplicate. In general, a two-fold increase in the mutation ratio (MR = mutant colonies on test plate/spontaneous mutant colonies on negative control plate) and a dose-response relationship observed in a test sample is considered a positive mutagenic response^33^,^34^.

### Statistical analysis

All data are reported as the mean with standard deviation. Statistical analyses were performing using SPSS for Windows software version 12.0 (SPSS Inc., Chicago, IL, USA). Comparisons between the group with and without S9 and the difference between the mutagenicity of the samples from Plant A and Plant B were analyzed using a paired sample *t*-test. Spearman correlation coefficients were used to describe the correlations between the parameters and mutagenicity. A one-way ANOVA was applied to determine the difference between seasons, and the water samples were divided into three parts according to hydrological periods: the level period, wet period, and dry period.

All reported *P* values are 2-sided, and *P* < 0.05 was considered to be statistically significant.

## Results

### Mutagenicity of drinking water from Plant A

According to the Ames test results, the water samples from Plant A demonstrated mutagenicity toward strain TA98, and this mutagenicity became stronger as the dose increased from 0.5 to 2.0 L/plate. Conversely, mutagenicity toward strain TA100 was only detected for the finished water (2 L/plate) from the 2009 level period (Table 1 and Table 2). The mutagenic strength decreased with S9 incubation in most samples except for the following: the distribution water (1 L/plate) from the 2007 level period, distribution water (2 L/plate) from the 2008 wet period, raw water (2 L/plate) from the 2009 dry period, and distribution water (2 L/plate) from the 2009 dry period.

### Mutagenicity of drinking water from Plant B

The Ames test results for the water samples from Plant B were similar to those from Plant A. The Ames test with TA100 was negative except for the distribution water (2 L/plate) from the 2009 dry period, whereas the 2 L/plate with TA98 presented mutagenicity (Table 3 and Table 4). Similar to the samples from Plant A, mutagenicity decreased with S9 incubation, except for the finished water (0.5, 1, and 2 L/plate) from the 2007 level period, raw water (2 L/plate) from the 2008 level and wet periods, and raw water and distribution water (1 and 2 L/plate) from the 2009 dry period.

## Discussion

### Comparison of the mutagenicity of drinking water from Plant A and Plant B

According to the results of the Ames test, mutagenicity decreased with S9 incubation in most of the samples. The paired sample *t*-test confirmed the existence of a significant difference between S9 activation and no S9 activation (based on the MR values of 2 L/plate (TA98), *α* = 0.05; Plant A: *t* = 4.034, *P* = 0.0004; Plant B: *t* = 2.458, *P* = 0.021). This finding indicated that the organic compounds in the water can be partly inactivated or degraded into less toxic substances but cannot be completely eliminated. The organic compounds in the water samples were mainly direct mutagens, though some indirect types may also be present^33^,^35^. Positive results were found for all of the TA98 (without S9) tests as the dose increased to 2 L/plate, whereas few TA100 tests (with or without S9 incubation) showed mutagenicity, which suggests that the organic compounds in the water were mainly frame-shift mutagens. This finding is consistent with previous reports^36^,^37^.

**Figure 2:**
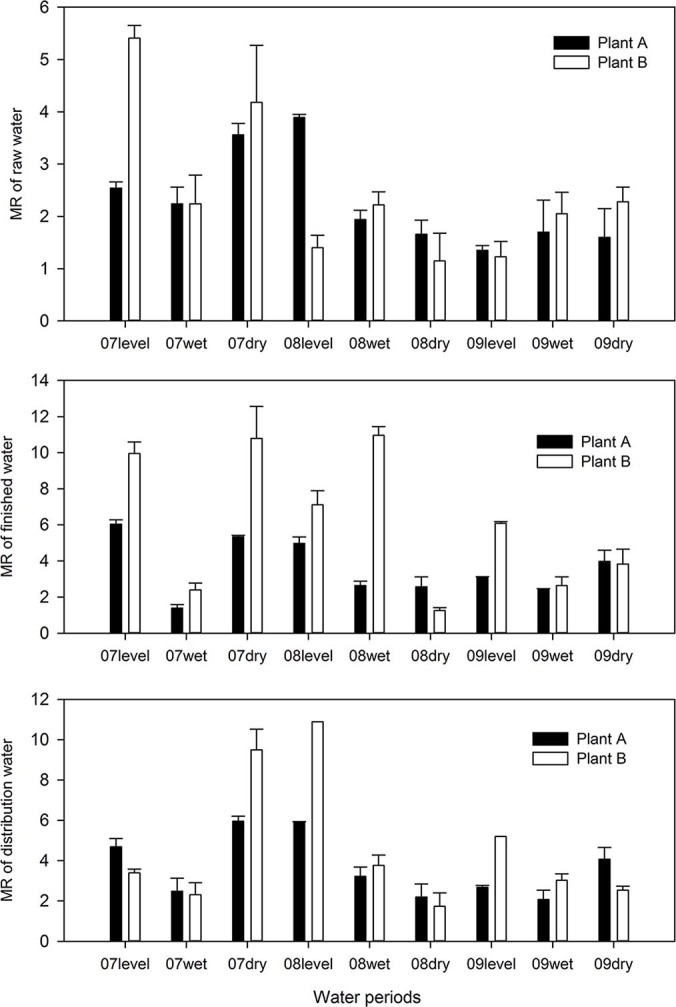
MR Values (mean ± deviation; TA98, −S9, 2 L) of Water Samples from Plant A and Plant B from Different Periods. MR: mutation ratio, the fold-increase over the negative control plates.

As shown in Supplementary Table S4, there was no significant difference between the distribution water and raw water from the two rivers. However, the mutagenicity between the finished waters was different, which may be due to the different chemical reactions in the treatment processes and the long water transportation pipelines, leading to the transformation of the organic compounds that were present in the water. As shown in Figure 2, the mutagenicity of the finished water samples was stronger in the samples from Plant B than in the samples from Plant A.

In the present study, genotoxicity was not detected in the raw water samples or was weaker than the corresponding finished water samples, as the finished water samples had higher MR values. This finding indicates that the DBPs form and genotoxicity emerges during the treatment process, which is in accordance with previous research^4^. And most of the distribution water presented weaker mutagenicity compared to the relative finished water. One possible explanation for this would be the long transportation (Plant A 3 km pipelines, Plant B 8 km pipelines) that cause change of the physical and chemical factors and the microorganisms in the pipeline that cause the degradation of the organic compounds^38^,^39^.

Compared to other studies in China^11^,^12^,^40^,^41^, the mutagenicity of Hanshui River samples was higher, with a maximum MR value of 10.96 (Table 3), whereas the mutagenicity of samples from the Yangtze River was average. In addition to the measured deviations, the large amount of sewage outfalls from the relatively concentrated industrial areas of Wuhan City and the residential activities along the river may be responsible for the high MR values of the Hanshui River samples.

### Seasonal-dependent variation of mutagenicity of water samples from Plant A and Plant B

As illustrated in Figure 2, the mutagenicity of the water samples varied during different periods. For Plant A, the mutagenicity strength was the strongest in 2007, followed by 2009. Furthermore, the results show some variation throughout a given year, with the lowest reactivity in the wet period, increasing in the dry period, and usually reaching a maximum in the level period. For Plant B, the mutagenicity strength was the strongest in 2008, followed by 2007; all of the wet periods in the three years presented the weakest mutagenicity strength, similar to Plant A.

As shown in Supplementary Table S5, only finished water from Plant A showed a significant difference in mutagenicity. As mentioned above, the wet period samples from both Plant A and Plant B showed the weakest mutagenicity strength, followed by samples from the dry period. Several factors may explain this finding, such as aquatic organisms, rainfall, runoff, and temperature in the various hydrological periods. Similarly, the activities of residents living along the river vary with the season. Jianyong Wu et al. reported that Taihu Lake water samples obtained in winter had the strongest mutability, which is consistent with our study^15^.

### Correlations between water quality parameters and the mutagenicity of water samples

**Figure 3:**
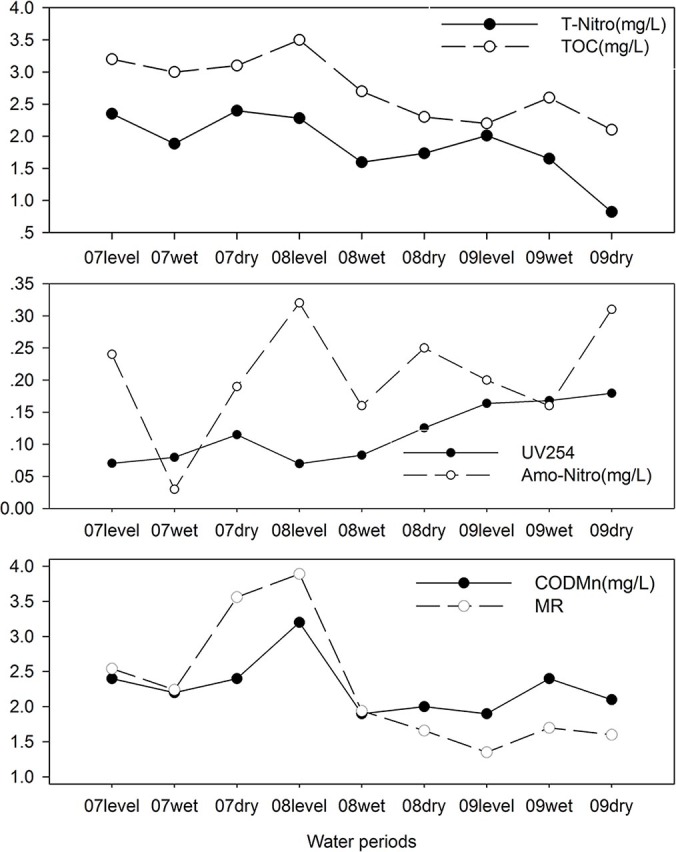
Water Parameters and MR Values (TA98, −S9, 2 L) of Raw Water from Plant A in Different Periods. MR: mutation ratio, the fold-increase over the negative control plates.

Raw water contains a large amount of humic acid, fulvic acid, and other organic materials, resulting in a high COD value. Chlorination, a widely applied technology to purify water, can lead to the oxidation of organic matter to decrease COD values, but the DBPs formed during chlorination lead to increased mutagenicity, as shown in this study. Although most carbon exists in the form of organic materials in water, the TOC reflects the organic pollution level. In general, as the concentration of TOC in raw water increases, the concentration of DBPs also increases^42^. Research has noted that there are correlations between water parameters and genotoxicity^43^,^44^,^45^. According to the quantity of water an adult consumes each day (suggested dose = 2 L/day, U.S. EPA^46^), the values of MR varied according to the trend of COD and other parameters, as illustrated in Figure 3. By applying Spearman correlations, the associations between mutagenicity and water quality parameters were determined. As shown in Supplementary Table S6, of the raw water samples, MR (−S9) had a positive correlation with COD, total nitrogen, and TOC. No correlations were found between MR (−S9) and the other parameters, and MR (+S9) had no positive correlation. As shown in Supplementary Table S7 and Table S8, only the MR values (+S9) of the distribution water samples showed a positive correlation with UV_254_.

The levels of mutagenicity produced by chlorination of the organic matter in natural waters was also found to rise with increased TOC^45^,^47^. Consistent with these observations, there was a fairly good correlation (*r* = 0.681) between the TOC concentration and the mutagenicity level in the raw water samples in our work. UV_254_ is the ultraviolet 254 nm absorption value. As water typically contains organic matter with benzene rings, phenolic hydroxyl groups, conjugated double bonds, and hydrophobic groups that have a high absorption peak (UV_254_ nm), UV_254_ indirectly reflects this type of organic matter in water. TOC is an indicator of the mass of organic matter, whereas UV_254_ accounts for the specific structure and functional groups^48^,^49^. Schenck et al. (2009) reported that chlorinated drinking water in a test with TA100 without S9 correlated well with TOC, with the correlation coefficient between finished water and the total organic halogen content being the highest, approximately 0.95^50^, which is in agreement with our results. The total nitrogen content of the water correlated well with the mutagenicity, whereas ammonia nitrogen was not correlated; thus, we can deduce that nitrate nitrogen plays a major role in mutagenicity, which is in agreement with a previous study^5^,^51^. Additionally, researchers have reported that strong correlations exist between genotoxicity and the UV_254_ of finished water^44^,^49^; however, correlations were not found in the present study.

The aquatic system and water treatment processes are variable, and river water quality is affected by many complicated factors, such as hydroclimatic factors and human activities. The correlations observed, therefore, require further investigation to have a wider application.

## Conclusion

With regard to the river environment, only a few studies have focused on the mutagenicity of water from the Yangtze River and Hanshui River. We measured the mutagenic potential of water from the two rivers, the major sources of drinking water in Central China, and demonstrated seasonal-dependent variations. The water parameters COD (*r* = 0.599, *P* = 0.009), TOC (*r* = 0.681, *P* = 0.02), UV_254_ (*r* = 0.711, *P* = 0.001), and total nitrogen (*r* = 0.570, *P* = 0.014) presented strong correlations with mutagenicity (TA98) at 2.0 L/plate. Genotoxicity data (MR values of the Ames test (TA98) at the dose of 2 L/plate) are often not examined when assessing the quality of drinking water, but these values were frequently at significant levels during this investigation, which highlight the importance of monitoring them in drinking water. Therefore, it is recommended that genetic toxicology assays, such as the Ames test (2 L/plate), be used as a routine measurement in water environment monitoring. The results from this field study of drinking water in Wuhan indicate the need for improvement in the standard of drinking water quality in China. Further studies on the effects of water from these rivers, including an epidemiological study of the people who drink from or live along them, and models that can predict genotoxicity using water parameters are needed.

## Supplementary Material

Supplementary Information Supplementary information

## Figures and Tables

**Table 1:** Results (MR: mean ± deviation) of the Ames Test (TA98) for Plant A

Period		Dose (L/plate)	2007		2008		2009	
			−S9	+S9	−S9	+S9	−S9	+S9
Level period	Raw water	0.5	1.35 ± 0.04	1.40 ± 0.16	1.44 ± 0.02	1.10 ± 0.13	0.65 ± 0.37	0.87 ± 0.08
		1	1.73 ± 0.00	1.73 ± 0.24	**2.46 ± 0.13**	1.43 ± 0.38	1.05 ± 0.17	0.85 ± 0.37
		2	**2.54 ± 0.12**	**2.22 ± 0.19**	**3.89 ± 0.06**	**2.11 ± 0.39**	1.35 ± 0.09	1.05 ± 0.42
	Finished water	0.5	**2.03 ± 0.07**	**2.08 ± 0.12**	**2.46 ± 0.26**	1.61 ± 0.21	1.63 ± 0.39	1.04 ± 0.03
		1	**2.86 ± 0.19**	**3.18 ± 0.47**	**3.12 ± 0.49**	1.77 ± 0.39	**2.17 ± 0.15**	1.28 ± 0.14
		2	**6.03 ± 0.25**	**4.20 ± 0.34**	**4.97 ± 0.36**	1.96 ± 0.35	**3.07 ± 0.06**	1.69 ± 0.13
	Distribution water	0.5	**2.21 ± 0.10**	1.59 ± 0.11	1.82 ± 0.12	1.28 ± 0.00	1.13 ± 0.63	1.02 ± 0.05
		1	**3.55 ± 0.19**	**2.24 ± 0.14**	**2.60 ± 0.62**	1.65 ± 0.06	1.87 ± 0.11	1.34 ± 0.21
		2	**4.69 ± 0.41**	**3.98 ± 0.02**	**5.93 ± 0.01**	**2.61 ± 0.45**	**2.68 ± 0.09**	1.73 ± 0.05
Wet period	Raw water	0.5	1.21 ± 0.66	1.25 ± 0.13	1.14 ± 0.14	1.09 ± 0.40	1.42 ± 0.33	0.99 ± 0.34
		1	1.62 ± 0.46	1.40 ± 0.22	1.42 ± 0.59	1.35 ± 0.35	1.58 ± 0.30	1.32 ± 0.17
		2	**2.24 ± 0.32**	1.80 ± 0.12	1.94 ± 0.18	1.95 ± 0.36	1.70 ± 0.61	1.65 ± 0.32
	Finished water	0.5	1.58 ± 0.24	1.12 ± 0.30	1.50 ± 0.44	1.23 ± 0.16	1.12 ± 0.65	1.49 ± 0.22
		1	1.92 ± 0.55	1.46 ± 0.15	1.90 ± 0.53	0.96 ± 0.31	1.51 ± 0.43	1.34 ± 0.25
		2	1.39 ± 0.20	1.68 ± 0.15	**2.64 ± 0.24**	**2.56 ± 0.20**	**2.40 ± 0.07**	1.72 ± 0.10
	Distribution water	0.5	1.54 ± 0.42	1.36 ± 0.29	1.28 ± 0.53	1.19 ± 0.40	1.43 ± 0.33	1.22 ± 0.40
		1	**2.06 ± 0.58**	1.44 ± 0.15	1.75 ± 0.59	1.35 ± 0.04	1.68 ± 0.60	1.22 ± 0.18
		2	**2.49 ± 0.64**	1.69 ± 0.13	**3.22 ± 0.47**	**2.21 ± 0.03**	**2.07 ± 0.46**	1.97 ± 0.28
Dry period	Raw water	0.5	**2.50 ± 0.47**	0.59 ± 0.29	1.45 ± 0.52	0.92 ± 0.40	1.08 ± 0.30	1.39 ± 0.36
		1	**2.23 ± 0.27**	0.68 ± 0.08	1.22 ± 0.38	1.16 ± 0.19	1.15 ± 0.13	1.52 ± 0.37
		2	**3.56 ± 0.22**	0.70 ± 0.56	1.66 ± 0.27	1.12 ± 0.27	1.60 ± 0.55	**2.54 ± 0.16**
	Finished water	0.5	**2.99 ± 0.20**	0.75 ± 0.21	1.14 ± 0.30	1.40 ± 0.09	1.29 ± 0.06	1.88 ± 0.43
		1	**2.18 ± 0.32**	0.89 ± 0.29	1.32 ± 0.46	1.21 ± 0.19	**2.27 ± 0.28**	**2.21 ± 0.31**
		2	**5.34 ± 0.08**	1.36 ± 0.15	**2.57 ± 0.55**	1.44 ± 0.17	**3.97 ± 0.62**	**2.67 ± 0.17**
	Distribution water	0.5	**3.66 ± 0.35**	0.92 ± 0.01	1.14 ± 0.54	1.00 ± 0.20	1.74 ± 0.51	**2.01 ± 0.19**
		1	**3.63 ± 0.55**	0.88 ± 0.08	1.61 ± 0.10	1.05 ± 0.39	**3.04 ± 0.29**	**2.82 ± 0.39**
		2	**5.96 ± 0.24**	1.62 ± 0.13	**2.20 ± 0.64**	1.30 ± 0.27	**4.07 ± 0.59**	**5.42 ± 0.40**

TA98, *Salmonella typhimurium* strain used in the Ames test to detect frame-shift mutants.

MR: mutation ratio, the fold-increase over the negative control plates.

**Table 2:** Results (MR: mean ± deviation) of the Ames test (TA100) for Plant A

Period		Dose (L/plate)	2007		2008		2009	
			−S9	+S9	−S9	+S9	−S9	+S9
Level period	Raw water	0.5	0.97 ± 0.02	0.96 ± 0.01	1.06 ± 0.06	0.94 ± 0.04	0.96 ± 0.12	0.98 ± 0.11
		1	1.25 ± 0.06	1.09 ± 0.03	1.02 ± 0.08	1.06 ± 0.06	1.07 ± 0.12	0.99 ± 0.11
		2	0.54 ± 0.02	1.13 ± 0.03	1.05 ± 0.21	1.01 ± 0.02	0.99 ± 0.22	1.11 ± 0.13
	Finished water	0.5	0.77 ± 0.00	1.08 ± 0.07	0.88 ± 0.13	0.79 ± 0.03	1.63 ± 0.11	0.97 ± 0.04
		1	0.75 ± 0.02	1.14 ± 0.04	0.85 ± 0.05	0.70 ± 0.09	1.39 ± 0.22	1.06 ± 0.14
		2	0.38 ± 0.01	1.46 ± 0.01	1.20 ± 0.01	0.51 ± 0.10	**2.08 ± 0.06**	1.21 ± 0.11
	Distribution water	0.5	0.86 ± 0.07	1.09 ± 0.05	1.07 ± 0.10	0.99 ± 0.05	1.05 ± 0.09	0.99 ± 0.06
		1	0.87 ± 0.07	1.25 ± 0.05	1.20 ± 0.12	1.07 ± 0.12	1.18 ± 0.15	0.90 ± 0.10
		2	0.35 ± 0.01	1.45 ± 0.07	1.50 ± 0.18	1.27 ± 0.10	1.46 ± 0.15	1.19 ± 0.06
Wet period	Raw water	0.5	1.33 ± 0.16	1.05 ± 0.07	1.04 ± 0.10	0.92 ± 0.03	1.10 ± 0.08	1.01 ± 0.04
		1	1.01 ± 0.26	0.96 ± 0.15	1.03 ± 0.13	0.90 ± 0.13	1.04 ± 0.00	0.93 ± 0.05
		2	1.28 ± 0.36	1.19 ± 0.09	0.93 ± 0.13	0.89 ± 0.06	1.14 ± 0.18	1.04 ± 0.01
	Finished water	0.5	1.17 ± 0.33	1.02 ± 0.09	1.16 ± 0.03	0.84 ± 0.04	1.01 ± 0.17	0.96 ± 0.08
		1	1.14 ± 0.29	1.11 ± 0.02	1.21 ± 0.01	0.84 ± 0.14	1.12 ± 0.09	1.05 ± 0.04
		2	1.05 ± 0.05	1.27 ± 0.06	1.34 ± 0.06	0.96 ± 0.05	1.27 ± 0.04	1.07 ± 0.00
	Distribution water	0.5	1.96 ± 0.07	1.09 ± 0.09	1.22 ± 0.07	0.80 ± 0.07	1.06 ± 0.22	1.14 ± 0.11
		1	1.79 ± 0.10	0.93 ± 0.08	1.07 ± 0.03	0.98 ± 0.02	1.06 ± 0.03	0.98 ± 0.04
		2	1.09 ± 0.18	1.15 ± 0.10	1.42 ± 0.19	1.00 ± 0.11	1.25 ± 0.21	1.16 ± 0.09
Dry period	Raw water	0.5	0.88 ± 0.10	1.05 ± 0.08	1.16 ± 0.02	0.84 ± 0.11	1.06 ± 0.13	1.09 ± 0.09
		1	0.89 ± 0.12	1.00 ± 0.05	1.00 ± 0.21	0.85 ± 0.08	0.99 ± 0.13	1.12 ± 0.05
		2	0.92 ± 0.03	1.13 ± 0.03	0.93 ± 0.09	0.92 ± 0.13	1.87 ± 0.01	1.23 ± 0.10
	Finished water	0.5	0.94 ± 0.07	1.06 ± 0.06	1.24 ± 0.04	0.79 ± 0.11	1.24 ± 0.14	0.99 ± 0.07
		1	1.01 ± 0.02	1.01 ± 0.08	1.02 ± 0.01	0.89 ± 0.14	1.29 ± 0.01	1.14 ± 0.03
		2	1.07 ± 0.05	1.05 ± 0.11	0.98 ± 0.02	1.06 ± 0.05	1.95 ± 0.04	1.29 ± 0.02
	Distribution water	0.5	1.00 ± 0.05	1.04 ± 0.15	1.46 ± 0.15	0.93 ± 0.09	1.14 ± 0.11	1.10 ± 0.11
		1	1.09 ± 0.09	1.06 ± 0.06	0.93 ± 0.05	0.77 ± 0.02	1.38 ± 0.23	1.24 ± 0.04
		2	1.41 ± 0.24	1.17 ± 0.10	0.88 ± 0.00	0.88 ± 0.10	1.63 ± 0.12	1.84 ± 0.13

TA100, *Salmonella typhimurium* strain used in the Ames test to detect base-replacement mutants.

MR: mutation ratio, the fold-increase over the negative control plates.

**Table 3:** Results (MR: mean ± deviation) of the Ames test (TA98) for Plant B

Period		Dose (L/plate)	2007		2008		2009	
			−S9	+S9	−S9	+S9	−S9	+S9
Level period	Raw water	0.5	1.71 ± 0.10	1.30 ± 0.16	**2.26 ± 0.74**	1.51 ± 0.17	1.04 ± 0.41	1.10 ± 0.10
		1	**2.48 ± 0.17**	**2.09 ± 0.44**	**2.61 ± 0.11**	**2.62 ± 0.50**	0.99 ± 0.48	1.25 ± 0.13
		2	**5.41 ± 0.24**	**3.35 ± 0.29**	1.40 ± 0.24	**4.73 ± 0.19**	1.23 ± 0.29	1.84 ± 0.23
	Finished water	0.5	1.99 ± 0.12	**3.03** ± **0.16**	**3.08** ± **0.50**	1.65 ± 0.19	**2.45 ± 0.10**	1.54 ± 0.11
		1	**3.41 ± 0.29**	**4.80 ± 0.31**	**3.64 ± 0.04**	**2.34 ± 0.13**	**3.50 ± 0.34**	**2.68 ± 0.55**
		2	**9.95 ± 0.65**	**10.1 ± 0.31**	**7.11 ± 0.79**	**3.73 ± 0.47**	**6.08 ± 0.09**	**5.05 ± 0.21**
	Distribution water	0.5	1.48 ± 0.05	1.38 ± 0.19	**2.70 ± 0.18**	1.77 ± 0.02	1.90 ± 0.70	1.82 ± 0.20
		1	1.94 ± 0.13	1.79 ± 0.46	**4.05 ± 0.55**	**2.86 ± 0.07**	**3.33 ± 0.31**	**2.96 ± 0.25**
		2	**3.39 ± 0.19**	**2.52 ± 0.31**	**10.89 ± 0.00**	**4.32 ± 0.51**	**5.20 ± 0.00**	**5.21 ± 0.23**
Wet period	Raw water	0.5	0.85 ± 0.17	1.21 ± 0.15	1.10 ± 0.76	1.44 ± 0.44	1.28 ± 0.69	1.09 ± 0.42
		1	1.00 ± 0.03	1.32 ± 0.05	1.68 ± 0.20	1.58 ± 0.34	1.40 ± 0.82	1.48 ± 0.51
		2	**2.24 ± 0.55**	1.92 ± 0.07	**2.22 ± 0.25**	**3.28 ± 0.23**	**2.05 ± 0.41**	1.76 ± 0.25
	Finished water	0.5	1.35 ± 0.49	1.10 ± 0.08	**3.90 ± 0.71**	**2.19 ± 0.47**	1.51 ± 0.54	1.64 ± 0.40
		1	1.91 ± 0.21	1.23 ± 0.06	**5.89 ± 0.29**	**2.89 ± 0.25**	1.85 ± 0.35	1.50 ± 0.22
		2	**2.39 ± 0.38**	1.47 ± 0.10	**10.96 ± 0.49**	**7.19 ± 0.48**	**2.63 ± 0.49**	**2.42 ± 0.02**
	Distribution water	0.5	1.43 ± 0.59	1.39 ± 0.11	1.70 ± 0.87	1.27 ± 0.29	1.42 ± 0.09	1.26 ± 0.47
		1	1.66 ± 0.56	1.51 ± 0.19	**2.94 ± 0.42**	1.97 ± 0.02	1.86 ± 0.33	1.62 ± 0.25
		2	**2.31 ± 0.59**	**2.10 ± 0.37**	**3.77 ± 0.51**	**3.15 ± 0.04**	**3.02 ± 0.33**	**2.39 ± 0.60**
Dry period	Raw water	0.5	**3.06 ± 0.17**	0.77 ± 0.30	1.08 ± 0.29	0.71 ± 0.12	1.26 ± 0.06	1.89 ± 0.22
		1	**2.23 ± 0.29**	0.82 ± 0.15	0.93 ± 0.33	0.77 ± 0.44	1.42 ± 0.28	**2.43 ± 0.05**
		2	**4.18 ± 1.09**	1.68 ± 0.16	1.15 ± 0.53	0.81 ± 0.09	**2.28 ± 0.28**	**3.50 ± 0.58**
	Finished water	0.5	**3.16 ± 0.42**	1.71 ± 0.14	1.01 ± 0.31	1.05 ± 0.05	1.63 ± 0.25	1.88 ± 0.51
		1	**5.15 ± 0.41**	2.17 ± 0.27	1.21 ± 0.23	0.95 ± 0.30	**2.55 ± 0.69**	**2.29 ± 0.32**
		2	**10.80 ± 1.76**	**4.77 ± 0.62**	1.25 ± 0.17	1.30 ± 0.09	**3.83 ± 0.82**	**3.82 ± 0.24**
	Distribution water	0.5	**3.82 ± 0.62**	1.50 ± 0.06	1.29 ± 0.02	1.08 ± 0.12	1.26 ± 0.73	1.65 ± 0.29
		1	**4.97 ± 1.84**	1.92 ± 0.11	1.58 ± 0.54	1.08 ± 0.27	1.93 ± 0.01	**2.06 ± 0.43**
		2	**9.50 ± 1.02**	**3.93 ± 0.60**	1.73 ± 0.67	1.23 ± 0.47	**2.54 ± 0.19**	**2.95 ± 0.13**

MR: mutation ratio, the fold-increase over the negative control plates.

**Table 4:** Results (MR: mean ± deviation) of the Ames test (TA100) for Plant B

Period		Dose (L/plate)	2007		2008		2009	
			−S9	+S9	−S9	+S9	−S9	+S9
Level period	Raw water	0.5	1.10 ± 0.04	1.09 ± 0.06	0.80 ± 0.06	1.02 ± 0.04	1.61 ± 0.11	1.15 ± 0.11
		1	1.12 ± 0.04	1.13 ± 0.10	0.73 ± 0.17	1.11 ± 0.18	1.19 ± 0.03	1.10 ± 0.17
		2	0.46 ± 0.02	1.38 ± 0.07	0.49 ± 0.00	1.32 ± 0.03	1.22 ± 0.12	1.31 ± 0.10
	Finished water	0.5	1.05 ± 0.03	1.09 ± 0.11	1.08 ± 0.16	0.88 ± 0.04	1.72 ± 0.18	1.12 ± 0.05
		1	1.67 ± 0.04	1.17 ± 0.09	1.07 ± 0.01	1.02 ± 0.11	1.12 ± 0.20	1.04 ± 0.13
		2	0.37 ± 0.01	1.75 ± 0.10	1.33 ± 0.11	1.15 ± 0.02	1.41 ± 0.11	1.34 ± 0.03
	Distribution water	0.5	1.08 ± 0.04	0.92 ± 0.07	1.10 ± 0.14	1.02 ± 0.11	1.01 ± 0.14	1.11 ± 0.04
		1	0.99 ± 0.06	0.83 ± 0.02	1.27 ± 0.02	1.17 ± 0.01	1.28 ± 0.04	1.15 ± 0.12
		2	0.45 ± 0.01	1.00 ± 0.03	1.40 ± 0.08	1.07 ± 0.16	1.75 ± 0.19	1.35 ± 0.01
Wet period	Raw water	0.5	1.03 ± 0.08	0.99 ± 0.05	1.11 ± 0.01	0.97 ± 0.18	1.11 ± 0.09	1.10 ± 0.06
		1	1.02 ± 0.25	1.05 ± 0.03	1.11 ± 0.06	0.93 ± 0.06	1.11 ± 0.20	1.04 ± 0.03
		2	0.81 ± 0.03	0.81 ± 0.22	1.16 ± 0.06	1.01 ± 0.02	1.36 ± 0.05	1.15 ± 0.10
	Finished water	0.5	0.95 ± 0.19	1.05 ± 0.02	1.12 ± 0.03	1.04 ± 0.11	1.20 ± 0.10	0.94 ± 0.12
		1	1.00 ± 0.06	1.12 ± 0.19	1.44 ± 0.13	1.10 ± 0.10	1.22 ± 0.09	1.05 ± 0.07
		2	1.01 ± 0.13	1.20 ± 0.13	1.62 ± 0.08	1.18 ± 0.05	1.28 ± 0.03	1.14 ± 0.06
	Distribution water	0.5	0.90 ± 0.14	1.03 ± 0.09	1.03 ± 0.18	1.07 ± 0.04	0.99 ± 0.23	1.04 ± 0.15
		1	0.96 ± 0.15	1.15 ± 0.08	1.06 ± 0.16	1.10 ± 0.14	1.08 ± 0.21	1.06 ± 0.17
		2	1.24 ± 0.11	1.03 ± 0.09	1.27 ± 0.05	0.98 ± 0.13	1.27 ± 0.02	1.19 ± 0.10
Dry period	Raw water	0.5	0.91 ± 0.07	1.03 ± 0.18	0.96 ± 0.22	0.98 ± 0.11	1.13 ± 0.20	0.96 ± 0.10
		1	0.86 ± 0.09	1.07 ± 0.04	0.84 ± 0.07	0.80 ± 0.13	1.12 ± 0.03	1.15 ± 0.05
		2	1.02 ± 0.12	1.17 ± 0.10	0.80 ± 0.19	0.85 ± 0.05	1.28 ± 0.20	1.63 ± 0.13
	Finished water	0.5	0.90 ± 0.09	1.06 ± 0.11	0.98 ± 0.01	0.86 ± 0.05	1.18 ± 0.21	1.07 ± 0.04
		1	1.02 ± 0.16	1.08 ± 0.09	1.03 ± 0.01	0.96 ± 0.09	1.46 ± 0.03	1.20 ± 0.17
		2	1.07 ± 0.17	1.39 ± 0.08	0.96 ± 0.10	0.91 ± 0.08	1.80 ± 0.07	1.88 ± 0.11
	Distribution water	0.5	1.06 ± 0.15	1.05 ± 0.06	1.20 ± 0.07	0.91 ± 0.04	1.27 ± 0.02	1.17 ± 0.06
		1	1.15 ± 0.06	1.11 ± 0.14	1.06 ± 0.06	0.91 ± 0.15	1.48 ± 0.05	1.14 ± 0.09
		2	1.56 ± 0.02	1.30 ± 0.04	0.89 ± 0.17	0.99 ± 0.15	**2.18 ± 0.14**	1.42 ± 0.15

MR: mutation ratio, the fold-increase over the negative control plates.

## References

[b1] Gray N. F. in Microbiology of Waterborne Diseases 2^nd^ edn, (eds Percival S., , Yates M. V., , Williams D. W., , Chalmers R. M., & Gray N. F., eds. ) 537–569 (Academic Press, 2014).

[b2] Fawell J. K. in Disinfection By-Products in Drinking Water (eds Fielding M., , Farrimond M., eds. ) 157–164 (Woodhead Publishing, 1999).

[b3] Doederer K., Gernjak W., Weinberg H. S. & Farré M. J. Factors affecting the formation of disinfection by-products during chlorination and chloramination of secondary effluent for the production of high quality recycled water. Water Research 48, 218–228, 10.1016/j.watres.2013.09.034 (2014). 24095593

[b4] Richardson S. D. in Encyclopedia of Environmental Health (ed Nriagu J. O., ed. ) 110–136 (Elsevier, 2011).

[b5] Fan Z. *et al.* Characterization, DBPs formation, and mutagenicity of different organic matter fractions in two source waters. International Journal of Hygiene and Environmental Health 217, 300–306, 10.1016/j.ijheh.2013.07.002 (2014). 23896129

[b6] Liu J. & Zhang X. Comparative toxicity of new halophenolic DBPs in chlorinated saline wastewater effluents against a marine alga: Halophenolic DBPs are generally more toxic than haloaliphatic ones. Water Research 65, 64–72, 10.1016/j.watres.2014.07.024 (2014). 25090624

[b7] Chowdhury S., Rodriguez M. J. & Sadiq R. Disinfection byproducts in Canadian provinces: Associated cancer risks and medical expenses. Journal of Hazardous Materials 187, 574–584, 10.1016/j.jhazmat.2011.01.085 (2011). 21292392

[b8] Ileka-Priouzeau S. *et al.* Women exposure during pregnancy to haloacetaldehydes and haloacetonitriles in drinking water and risk of small-for-gestational-age neonate. Environmental Research 137, 338–348, 10.1016/j.envres.2015.01.005 (2015). 25601737

[b9] Chowdhury S., Champagne P. & McLellan P. J. Uncertainty characterization approaches for risk assessment of DBPs in drinking water: a review. J Environ Manage 90, 1680–1691, 10.1016/j.jenvman.2008.12.014 (2009). 19167150

[b10] Ye Y. *et al.* Assessing of genotoxicity of 16 centralized source-waters in China by means of the SOS/umu assay and the micronucleus test: Initial identification of the potential genotoxicants by use of a GC/MS method and the QSAR Toolbox 3.0. Mutation Research/Genetic Toxicology and Environmental Mutagenesis 763, 36–43, 10.1016/j.mrgentox.2013.11.003 (2014). 24525378

[b11] Ren H. *et al.* Continuous surface seawater surveillance on poly aromatic hydrocarbons (PAHs) and mutagenicity of East and South China Seas. Estuarine, Coastal and Shelf Science 86, 395–400, 10.1016/j.ecss.2009.09.025 (2010).

[b12] Zhao Z., Zhang L., Wu J., Fan C. & Shang J. Assessment of the potential mutagenicity of organochlorine pesticides (OCPs) in contaminated sediments from Taihu Lake, China. Mutat Res 696, 62–68, 10.1016/j.mrgentox.2009.12.013 (2010). 20036756

[b13] Yuan J. *et al.* Chlorinated river and lake water extract caused oxidative damage, DNA migration and cytotoxicity in human cells. Int J Hyg Environ Health 208, 481–488, 10.1016/j.ijheh.2005.09.002 (2005). 16325558

[b14] Liu S., Zhu Z., Fan C., Qiu Y. & Zhao J. Seasonal variation effects on the formation of trihalomethane during chlorination of water from Yangtze River and associated cancer risk assessment. J Environ Sci (China) 23, 1503–1511 (2011). 22432287 10.1016/s1001-0742(10)60573-6

[b15] Wu J. Y. *et al.* A season-dependent variation of genotoxicity of surface water samples from Taihu Lake, Yangzte delta. Environ Monit Assess 98, 225–234 (2004). 15473538 10.1023/b:emas.0000038188.16088.f3

[b16] Kolkman A. *et al.* Sample preparation for combined chemical analysis and in vitro bioassay application in water quality assessment. Environmental Toxicology and Pharmacology 36, 1291–1303, 10.1016/j.etap.2013.10.009 (2013). 24216068

[b17] Smital T. *et al.* Assessment of toxicological profiles of the municipal wastewater effluents using chemical analyses and bioassays. Ecotoxicology and Environmental Safety 74, 844–851, 10.1016/j.ecoenv.2010.11.010 (2011). 21159381

[b18] Wharfe J. Hazardous chemicals in complex mixtures--a role for direct toxicity assessment. Ecotoxicology 13, 413–421 (2004). 15462133 10.1023/b:ectx.0000035292.00099.f0

[b19] Russo R. C. Development of marine water quality criteria for the USA. Mar Pollut Bull 45, 84–91 (2002). 12398371 10.1016/s0025-326x(02)00136-4

[b20] Chowdhury S., Rodriguez M. J., Sadiq R. & Serodes J. Modeling DBPs formation in drinking water in residential plumbing pipes and hot water tanks. Water Research 45, 337–347, 10.1016/j.watres.2010.08.002 (2011). 20732706

[b21] Sadiq R. & Rodriguez M. J. in Encyclopedia of Environmental Health (ed Nriagu J. O., ed. ) 282–295 (Elsevier, 2011).

[b22] Wang C. *et al.* Effects of organic fractions on the formation and control of N-nitrosamine precursors during conventional drinking water treatment processes. Science of The Total Environment 449, 295–301, 10.1016/j.scitotenv.2013.01.080 (2013). 23435061

[b23] American Public Health Association. . Standard methods for the examination of water and wastewater 21^st^ edn, (eds Rice E. W., , Baird R. B., , Eaton A. D., , Clesceri L. S., eds. ) (American Public Health Association, 2005).

[b24] Vega-Morales T., Sosa-Ferrera Z. & Santana-Rodriguez J. J. Determination of alkylphenol polyethoxylates, bisphenol-A, 17alpha-ethynylestradiol and 17beta-estradiol and its metabolites in sewage samples by SPE and LC/MS/MS. J Hazard Mater 183, 701–711, 10.1016/j.jhazmat.2010.07.083 (2010). 20724070

[b25] Warren S. H. *et al.* Survey of the mutagenicity of surface water, sediments, and drinking water from the Penobscot Indian Nation. Chemosphere 120, 690–696, 10.1016/j.chemosphere.2014.10.002 (2015). 25462314

[b26] Leme D. M. *et al.* Genotoxicity assessment of water soluble fractions of biodiesel and its diesel blends using the Salmonella assay and the in vitro MicroFlow® kit (Litron) assay. Chemosphere 86, 512–520, 10.1016/j.chemosphere.2011.10.017 (2012). 22071371

[b27] Estévez J. & Vilanova E. in Encyclopedia of Toxicology 3^rd^ edn, (ed. Wexler P., ed. ) 885-890 (Academic Press, 2014).

[b28] Richardson S. D., Plewa M. J., Wagner E. D., Schoeny R. & Demarini D. M. Occurrence, genotoxicity, and carcinogenicity of regulated and emerging disinfection by-products in drinking water: a review and roadmap for research. Mutat Res 636, 178–242, 10.1016/j.mrrev.2007.09.001 (2007). 17980649

[b29] Hrudey S. E. Chlorination disinfection by-products, public health risk tradeoffs and me. Water Research 43, 2057–2092, 10.1016/j.watres.2009.02.011 (2009). 19304309

[b30] Standards C. S. B. O. in Standards for drinking water quality Vol. GB 5749–2006 (China Standards Press, Beijing, 2007).

[b31] Ames B. N., McCann J. & Yamasaki E. Methods for detecting carcinogens and mutagens with the Salmonella/mammalian-microsome mutagenicity test. Mutat Res 31, 347–364 (1975). 768755 10.1016/0165-1161(75)90046-1

[b32] Maron D. M. & Ames B. N. Revised methods for the Salmonella mutagenicity test. Mutat Res 113, 173–215 (1983). 6341825 10.1016/0165-1161(83)90010-9

[b33] Sujbert L. *et al.* Genotoxic potential of by-products in drinking water in relation to water disinfection: Survey of pre-ozonated and post-chlorinated drinking water by Ames-test. Toxicology 219, 106–112, 10.1016/j.tox.2005.11.015 (2006). 16364533

[b34] Guzzella L., Feretti D. & Monarca S. Advanced oxidation and adsorption technologies for organic micropollutant removal from lake water used as drinking-water supply. Water Research 36, 4307–4318, 10.1016/S0043-1354(02)00145-8 (2002). 12420936

[b35] Filipic M. Mutagenicity and toxicity of water extracts from the Sora river area. Mutat Res 342, 1–8 (1995). 7885390 10.1016/0165-1218(95)90084-5

[b36] Ohe T., Watanabe T. & Wakabayashi K. Mutagens in surface waters: a review. Mutat Res 567, 109–149, 10.1016/j.mrrev.2004.08.003 (2004). 15572284

[b37] Liu J.-R. *et al.* Genotoxicity of water from the Songhua River, China, in 1994–1995 and 2002–2003: Potential risks for human health. Environmental pollution 157, 357–364, 10.1016/j.envpol.2008.10.004 (2009). 19027211

[b38] Maffei F. *et al.* Drinking water quality: An in vitro approach for the assessment of cytotoxic and genotoxic load in water sampled along distribution system. Environment International 35, 1053–1061, 10.1016/j.envint.2009.05.007 (2009). 19573924

[b39] Guzzella L. *et al.* Detection of mutagens in water-distribution systems after disinfection. Mutation Research/Genetic Toxicology and Environmental Mutagenesis 608, 72–81, 10.1016/j.mrgentox.2006.05.010 (2006). 16863700

[b40] Shen L. *et al.* The mutagenic potentials of tap water samples in Shanghai. Chemosphere 52, 1641–1646, 10.1016/S0045-6535(03)00504-6 (2003). 12867198

[b41] Zhang G. Mechanism study of the coagulant impact on mutagenic activity in water. Water Research 34, 1781–1790, 10.1016/s0043-1354(99)00332-2 (2000).

[b42] Pressman J. G. *et al.* Disinfection byproduct formation in reverse-osmosis concentrated and lyophilized natural organic matter from a drinking water source. Water Research 46, 5343–5354, 10.1016/j.watres.2012.07.020 (2012). 22846256

[b43] Chowdhury S., Champagne P. & McLellan P. J. Models for predicting disinfection byproduct (DBP) formation in drinking waters: a chronological review. The Science of the total environment 407, 4189–4206, 10.1016/j.scitotenv.2009.04.006 (2009). 19419751

[b44] Wang D., Xu Z., Zhao Y., Yan X. & Shi J. Change of genotoxicity for raw and finished water: role of purification processes. Chemosphere 83, 14–20, 10.1016/j.chemosphere.2011.01.039 (2011). 21315407

[b45] Vartiainen T., Liimatainen A., Kauranen P. & Hiisvirta L. Relations between drinking water mutagenicity and water quality parameters. Chemosphere 17, 189–202, 10.1016/0045-6535(88)90056-2 (1988).

[b46] US.EPA. . Risk Assessment Guidance for Superfund.vol. I, Human Health Evaluation Manual. Part B. Development of Risk-based Preliminary Remediation Goals (Interim), PB92-963333. Publication 9285.7-01B. 9285.7-01B edn, Vol. EPA/540/R-92/003 (1991b).

[b47] Meier J. R., Lingg R. D. & Bull R. J. Formation of mutagens following chlorination of humic acid. A model for mutagen formation during drinking water treatment. Mutat Res 118, 25–41 (1983). 6223225 10.1016/0165-1218(83)90113-1

[b48] Croué J.-P., Violleau D. & Labouyrie L. Disinfection By-Product Formation Potentials of Hydrophobic and Hydrophilic Natural Organic Matter Fractions: A Comparison Between a Low- and a High-Humic Water. Vol. 761 (American Chemical Society, 2000).

[b49] Kulkarni P. & Chellam S. Disinfection by-product formation following chlorination of drinking water: Artificial neural network models and changes in speciation with treatment. Science of The Total Environment 408, 4202–4210, 10.1016/j.scitotenv.2010.05.040 (2010). 20580059

[b50] Schenck K. M., Sivaganesan M. & Rice G. E. Correlations of water quality parameters with mutagenicity of chlorinated drinking water samples. Journal of toxicology and environmental health. Part A 72, 461–467, 10.1080/15287390802608940 (2009). 19267307

[b51] Wu J. Y. Assessing surface water quality of the Yangtze Estuary with genotoxicity data. Mar Pollut Bull 50, 1661–1667, 10.1016/j.marpolbul.2005.07.001 (2005). 16098541

