# Supplementary material for: Mutagenicity of drinking water sampled from the Yangtze River and Hanshui River (Wuhan section) and correlations with water quality parameters
Source: Sci Rep. 2015 Mar 31;5:9572. doi: 10.1038/srep09572 (PMC5380332; doi:10.1038/srep09572)
Supplement: Supplementary Information [file srep09572-s1.doc]

**Mutagenicity of drinking water sampled from the Yangtze River and Hanshui River (Wuhan section) and correlations with water quality parameters**

**About the authors**

**Xuemin Lv, Yi Lu, Xiaoming Yang, Xiaorong Dong, Kunpeng Ma, Sanhua Xiao, Yazhou Wang, Fei Tang***

Department of Environmental Microbiology, Institute of Environmental Medicine, MOE Key Lab of Environment and Health, School of Public Health, Tongji Medical College, Huazhong University of Science and Technology, Wuhan 430030, PR China

**Corresponding Author**

E-mail: feitang@mails.tjmu.edu.cn

**Supplementary information**

Table S1. Water quality parameters of raw water from Plant A and Plant B

| Period | Year | Raw water | COD_Mn_  (mg/L) | Temp.  (°C) | pH | Turbidity  (NTU) | Ammonia nitrogen  (mg/L) | TOC  (mg/L) | Total nitrogen  (mg/L) | UV_254_ |
| --- | --- | --- | --- | --- | --- | --- | --- | --- | --- | --- |
| Level period | 2007 | Plant A | 2.4 | 15.5 | 7.50 | 35.00 | 0.24 | 3.20 | 2.35 | 0.0705 |
|  |  | Plant B | 3.3 | 9.0 | 8.08 | 30.70 | 0.03 | 5.30 | 2.19 | 0.0685 |
|  | 2008 | Plant A | 3.2 | 15.0 | 7.77 | 40.00 | 0.32 | 3.50 | 2.28 | 0.0699 |
|  |  | Plant B | 3.1 | 16.0 | 8.07 | 27.80 | 0.36 | 2.80 | 1.89 | 0.0703 |
|  | 2009 | Plant A | 1.9 | 11.8 | 7.84 | 60.50 | 0.20 | 2.20 | 2.01 | 0.1637 |
|  |  | Plant B | 2.2 | 16.0 | 8.00 | 31.00 | 0.25 | 3.20 | 1.92 | 0.0938 |
| Wet period | 2007 | Plant A | 2.2 | 27.0 | 7.68 | 140.00 | 0.03 | 3.00 | 1.88 | 0.0796 |
|  |  | Plant B | 3.1 | 30.0 | 8.02 | 107.60 | 0.06 | 3.90 | 2.21 | 0.0534 |
|  | 2008 | Plant A | 1.9 | 28.5 | 7.80 | 50.60 | 0.16 | 2.70 | 1.60 | 0.0831 |
|  |  | Plant B | 2.2 | 28.0 | 8.25 | 6.80 | 0.00 | 3.10 | 1.76 | 0.0515 |
|  | 2009 | Plant A | 2.4 | 27.0 | 7.80 | 37.20 | 0.16 | 2.60 | 1.65 | 0.1680 |
|  |  | Plant B | 3.8 | 29.0 | 8.03 | 54.60 | 0.06 | 2.90 | 0.98 | 0.1883 |
| Dry period | 2007 | Plant A | 2.4 | 13.0 | 7.69 | 36.80 | 0.19 | 3.10 | 2.40 | 0.1149 |
|  |  | Plant B | 3.4 | 9.0 | 7.98 | 46.70 | 0.18 | 4.30 | 2.65 | 0.0631 |
|  | 2008 | Plant A | 2.0 | 12.5 | 7.80 | 37.90 | 0.25 | 2.30 | 1.73 | 0.1256 |
|  |  | Plant B | 2.7 | 10.0 | 7.99 | 46.90 | 0.03 | 2.30 | 1.42 | 0.0625 |
|  | 2009 | Plant A | 2.1 | 9.0 | 7.80 | 44.00 | 0.31 | 2.10 | 0.82 | 0.1795 |
|  |  | Plant B | 3.4 | 9.5 | 7.92 | 117.00 | 0.27 | 2.80 | 0.99 | 0.1031 |

Abbreviations: COD_Mn_, chemical oxygen demand, as assessed by potassium permanganate; Temp, temperature; UV_254_, ultraviolet 254 nm

Table S2. Water quality parameters of finished water from Plant A and Plant B

| Period | Year | Finished water | COD_Mn_  (mg/L) | Temp.  (°C) | pH | Residual chlorine  (mg/L) | TOC  (mg/L) | Nitrate (mg/L) | UV_254_ |
| --- | --- | --- | --- | --- | --- | --- | --- | --- | --- |
| Level period | 2007 | Plant A | 2.2 | 15.5 | 7.40 | 1.00 | 1.60 | 1.68 | 0.0135 |
|  |  | Plant B | 1.9 | 9.0 | 7.69 | 0.80 | 2.30 | 2.24 | 0.0270 |
|  | 2008 | Plant A | 2.0 | 15.0 | 7.53 | 1.06 | 1.10 | 1.15 | 0.0162 |
|  |  | Plant B | 1.7 | 16.0 | 7.71 | 0.70 | 1.90 | 0.79 | 0.0290 |
|  | 2009 | Plant A | 1.0 | 11.8 | 7.60 | 0.81 | 1.00 | 1.52 | 0.0239 |
|  |  | Plant B | 1.3 | 16.0 | 7.86 | 0.67 | 1.50 | 1.59 | 0.0248 |
| Wet period | 2007 | Plant A | 1.0 | 27.0 | 7.45 | 1.20 | 1.50 | 1.18 | 0.0314 |
|  |  | Plant B | 1.7 | 30.0 | 7.78 | 0.80 | 2.90 | 2.23 | 0.0317 |
|  | 2008 | Plant A | 1.0 | 28.5 | 7.58 | 1.00 | 1.20 | 1.13 | 0.0303 |
|  |  | Plant B | 1.3 | 28.0 | 7.81 | 0.93 | 2.30 | 1.15 | 0.0319 |
|  | 2009 | Plant A | 1.4 | 27.0 | 7.51 | 0.70 | 1.30 | 0.99 | 0.0221 |
|  |  | Plant B | 2.2 | 29.0 | 7.76 | 0.74 | 2.00 | 1.32 | 0.0367 |
| Dry period | 2007 | Plant A | 1.4 | 13.0 | 7.51 | 0.91 | 1.10 | 1.34 | 0.0351 |
|  |  | Plant B | 1.8 | 9.0 | 7.78 | 0.68 | 3.20 | 1.51 | 0.0430 |
|  | 2008 | Plant A | 1.1 | 12.5 | 7.50 | 0.80 | 1.20 | 1.18 | 0.0370 |
|  |  | Plant B | 1.5 | 10.0 | 7.89 | 0.80 | 1.40 | 1.17 | 0.0421 |
|  | 2009 | Plant A | 1.2 | 9.0 | 7.53 | 0.77 | 1.10 | 1.79 | 0.0148 |
|  |  | Plant B | 1.3 | 9.5 | 7.74 | 0.77 | 1.50 | 2.19 | 0.0316 |

Abbreviations: COD_Mn_, chemical oxygen demand, as assessed by potassium permanganate; Temp, temperature; TOC, total organic carbon; UV_254_, ultraviolet 254 nm

Table S3. Water quality parameters of distribution water from Plant A and Plant B

| Period | Year | Distribution water | COD_Mn_  (mg/L) | Temp.  (°C) | pH | Residual chlorine  (mg/L) | TOC  (mg/L) | Nitrate (mg/L) | UV_254_ |
| --- | --- | --- | --- | --- | --- | --- | --- | --- | --- |
| Level period | 2007 | Plant A | 2.0 | 15.5 | 7.83 | 0.50 | 1.50 | 1.69 | 0.0129 |
|  |  | Plant B | 2.1 | 9.0 | 7.95 | 0.50 | 2.30 | 2.25 | 0.0273 |
|  | 2008 | Plant A | 2.0 | 15.0 | 7.90 | 0.40 | 1.20 | 1.75 | 0.0131 |
|  |  | Plant B | 2.0 | 16.0 | 7.97 | 0.10 | 1.70 | 1.55 | 0.0312 |
|  | 2009 | Plant A | 1.1 | 11.8 | 7.85 | 0.40 | 1.10 | 1.41 | 0.0415 |
|  |  | Plant B | 1.4 | 16.0 | 7.76 | 0.40 | 1.50 | 1.55 | 0.0246 |
| Wet period | 2007 | Plant A | 2.4 | 27.0 | 8.30 | 0.30 | 1.70 | 1.62 | 0.0356 |
|  |  | Plant B | 2.3 | 30.0 | 8.03 | 0.10 | 2.90 | 2.40 | 0.0314 |
|  | 2008 | Plant A | 1.0 | 28.5 | 7.70 | 0.40 | 1.20 | 1.14 | 0.0307 |
|  |  | Plant B | 1.3 | 28.0 | 7.80 | 0.30 | 1.70 | 1.12 | 0.0276 |
|  | 2009 | Plant A | 1.3 | 27.0 | 7.65 | 0.40 | 1.40 | 1.17 | 0.0304 |
|  |  | Plant B | 1.9 | 29.0 | 7.71 | 0.50 | 2.30 | 1.35 | 0.0407 |
| Dry period | 2007 | Plant A | 1.9 | 13.0 | 7.92 | 0.50 | 1.20 | 1.34 | 0.0389 |
|  |  | Plant B | 2.0 | 9.0 | 8.18 | 0.50 | 2.00 | 1.54 | 0.0340 |
|  | 2008 | Plant A | 1.0 | 12.5 | 7.79 | 0.50 | 1.30 | 1.17 | 0.0392 |
|  |  | Plant B | 0.5 | 10.0 | 7.96 | 0.50 | 1.40 | 1.21 | 0.0412 |
|  | 2009 | Plant A | 1.1 | 9.0 | 7.75 | 0.50 | 1.20 | 1.81 | 0.0156 |
|  |  | Plant B | 1.2 | 9.5 | 7.83 | 0.50 | 1.60 | 2.19 | 0.0346 |

Abbreviations: COD_Mn_, chemical oxygen demand, as assessed by potassium permanganate; Temp, temperature; TOC, total organic carbon; UV_254_, ultraviolet 254 nm

Table S4. Paired sample *t*-test of the mutagenicity of water samples from Plant A and Plant B (*α*=0.05)

|  | *t* | *P* |
| --- | --- | --- |
| Finished water | -3.542 | 0.002* |
| Distribution water | -1.588 | 0.124 |
| Raw water | -.259 | 0.797 |

*P*, value of significance. N (sum of the samples) = 27.

* Significance at the 0.05 level (2-tailed).

Table S5. One-way ANOVA of the MR values of finished water and distribution water from Plant A and Plant B (*α*=0.05)

|  |  | Sum of Squares | df | Mean Square | *F* | *P* |
| --- | --- | --- | --- | --- | --- | --- |
| Raw water Plant A | Between Groups | 2.131 | 2 | 1.066 | 2.659 | 0.091 |
|  | Within Groups | 9.619 | 24 | 0.401 |  |  |
|  | Total | 11.750 | 26 |  |  |  |
| Raw water Plant B | Between Groups | 1.323 | 2 | 0.662 | 1.683 | 0.207 |
|  | Within Groups | 9.432 | 24 | 0.393 |  |  |
|  | Total | 10.755 | 26 |  |  |  |
| Finished water Plant A | Between Groups | 9.168 | 2 | 4.584 | 5.631 | 0.010* |
|  | Within Groups | 19.539 | 24 | 0.814 |  |  |
|  | Total | 28.707 | 26 |  |  |  |
| Finished water Plant B | Between Groups | 31.113 | 2 | 15.557 | 2.664 | 0.090 |
|  | Within Groups | 140.170 | 24 | 5.840 |  |  |
|  | Total | 171.283 | 26 |  |  |  |
| Distribution water Plant A | Between Groups | 2.535 | 2 | 1.267 | 1.128 | 0.340 |
|  | Within Groups | 26.969 | 24 | 1.124 |  |  |
|  | Total | 29.503 | 26 |  |  |  |
| Distribution water Plant B | Between Groups | 16.696 | 2 | 8.348 | 2.506 | 0.103 |
|  | Within Groups | 79.957 | 24 | 3.332 |  |  |
|  | Total | 96.653 | 26 |  |  |  |

*P*, value of significance. N (sum of the samples) = 27.

* Significance at the 0.05 level (2-tailed).

Table S6. Spearman correlation between MR values (TA98, 2 L/plate) and raw water parameters

|  |  | COD_mn_  (mg/L) | Temp.  (°C) | pH | Turbidity  (NTU) | Ammonia nitrogen  (mg/L) | Total nitrogen  (mg/L) | UV_254_ | TOC  (mg/L) |
| --- | --- | --- | --- | --- | --- | --- | --- | --- | --- |
| MR(-S9) | *r* | 0.599** | -0.108 | 0.047 | -0.241 | 0.093 | 0.570* | -0.39 | 0.681** |
|  | *P* | 0.009 | 0.669 | 0.854 | 0.336 | 0.714 | 0.014 | 0.109 | 0.02 |
| MR(+S9) | *r* | 0.175 | -0.026 | 0.149 | -0.067 | -0.002 | -0.135 | -0.22 | 0.351 |
|  | *P* | 0.486 | 0.919 | 0.554 | 0.791 | 0.993 | 0.593 | 0.381 | 0.154 |

Abbreviations: COD_Mn_, chemical oxygen demand, as assessed by potassium permanganate; Temp, temperature; UV_254_, ultraviolet 254 nm; TOC, total organic carbon

r, Spearman correlation coefficient; *P*, value of significance. N (sum of the samples) = 18.

* Significance at the 0.05 level (2-tailed).

** Significance at the 0.01 level (2-tailed).

Table S7. Spearman correlation between MR values (TA98, 2 L/plate) and finished water parameters

|  | | COD_mn_  (mg/L) | Temp.  (°C) | pH | Residual chlorine  (mg/L) | TOC  (mg/L) | Nitrate  (mg/L) | UV_254_ |
| --- | --- | --- | --- | --- | --- | --- | --- | --- |
| MR(-S9) | *r* | 0.293 | -0.284 | 0.184 | -0.170 | 0.318 | 0.129 | -0.152 |
|  | *P* | 0.239 | 0.254 | 0.465 | 0.499 | 0.198 | 0.610 | 0.548 |
| MR(+S9) | *r* | 0.236 | -0.146 | 0.267 | -0.270 | .0505^*^ | 0.242 | -0.286 |
|  | *P* | 0.347 | 0.563 | 0.285 | 0.278 | 0.032 | 0.334 | 0.250 |

Abbreviations: COD_Mn_, chemical oxygen demand, as assessed by potassium permanganate; Temp, temperature; TOC, total organic carbon; UV_254_, ultraviolet 254 nm;

r, Spearman correlation coefficient; *P*, value of significance. N (sum of the samples) = 18.

* Significance at the 0.05 level (2-tailed).

** Significance at the 0.01 level (2-tailed).

Table S8. Spearman correlation between MR values (TA98, 2 L/plate) and distribution water parameters

|  | | COD_mn_  (mg/L) | Temp.  (°C) | pH | Residual chlorine  (mg/L) | TOC  (mg/L) | Nitrate  (mg/L) | UV_254_ |
| --- | --- | --- | --- | --- | --- | --- | --- | --- |
| MR(-S9) | *r* | 0.358 | -0.170 | 0.161 | -0.006 | -0.014 | 0.167 | 0.457 |
|  | *P* | 0.145 | 0.501 | 0.523 | 0.982 | 0.957 | 0.507 | 0.056 |
| MR(+S9) | *r* | 0.219 | -0.112 | -0.151 | -0.057 | 0.207 | 0.384 | 0.711^**^ |
|  | *P* | 0.382 | 0.659 | 0.550 | 0.822 | 0.409 | 0.115 | 0.001 |

Abbreviations: COD_Mn_, chemical oxygen demand, as assessed by potassium permanganate; Temp, temperature; TOC, total organic carbon; UV_254_, ultraviolet 254 nm;

r, Spearman correlation coefficient; *p*, value of significance. N (sum of the samples) = 18.

* Significance at the 0.05 level (2-tailed).

** Significance at the 0.01 level (2-tailed).
